# Whole Grains and Consumer Understanding: Investigating Consumers’ Identification, Knowledge and Attitudes to Whole Grains

**DOI:** 10.3390/nu12082170

**Published:** 2020-07-22

**Authors:** Shara Foster, Eleanor Beck, Jaimee Hughes, Sara Grafenauer

**Affiliations:** 1 School of Medicine, University of Wollongong, Northfields Avenue, Wollongong 2522, Australia; sif087@uowmail.edu.au (S.F.); eleanor@uow.edu.au (E.B.); 2 Illawarra Health & Medical Research Institute, Northfields Avenue, Wollongong 2552, Australia; 3 Grains & Legumes Nutrition Council, 1 Rivett Rd, North Ryde 2113, Australia; j.hughes@glnc.org.au

**Keywords:** whole grain, grains, fibre, health benefits, education, food labelling

## Abstract

Whole grains may assist in reducing risk of non-communicable disease, but consumption is limited in many countries. In Australia, the reasons for poor consumption are not well understood. The aim of this study was to investigate consumers’ knowledge, attitudes and identification of whole grains, incorporating an exploration of factors influencing consumption, promotion and provision. An online semi-structured questionnaire was used to gather responses from 735 participants (61% complete responses). Although 92% of respondents consumed grains, only 8% reported an intake consistent with age and gender recommendations. Refined pasta and rice were the most frequently purchased grain foods followed by wholemeal/whole grain bread. Of whole grain foods, bread and breakfast cereals were consumed more frequently. However, overall, participants did not prioritise consumption of whole grains. Despite this, 93% of participants had seen food packaging information drawing attention to whole grain content, with a high proportion describing whole grain as less processed (72%) or high in dietary fibre (67%). Two-thirds were aware of health benefits but stated that if they had further information, they would be more likely to swap to whole grain. Further education, increasing exposure, accessibility and extensive promotion of whole grain health benefits are required to facilitate whole grain consumption. Furthermore, removing the negative stigma associated with carbohydrate foods, including grains, will be necessary to improve consumption.

## 1. Introduction

The body of evidence supporting whole grains continues to grow and highlights the significant health benefits of regular consumption and protective effects [1]. These effects include decreased risk of a range of chronic illnesses such as type 2 diabetes mellitus [2], cardiovascular disease [2,3], certain cancers including colorectal [3], along with their potential risk factors including obesity [2,4], hypertension and elevated fasting glucose, insulin and low-density lipoprotein (LDL) cholesterol levels [5]. It has been suggested that the health benefits of whole grain consumption may be attributed to the synergistic effects of the bran and germ component of whole grains, as they are rich in micronutrients, dietary fibre and phytochemicals [6,7]. However, the complete mechanisms as to how these constituents have such health benefits remains unclear. Results from the National Nutrition and Physical Activity Survey 2011–2012 (NNPAS) indicated that approximately 30% of Australians met recommended grain intake, with one-third (34%) of total grains consumed as whole grains. Whole grains made up 55% of all grains consumed by individuals over 71 years, which was double of those aged 14–18 years (22%) [8]. Consumption of whole grains was calculated at 21 g/d for adults (19–85 years) [9], indicating inadequate whole grain consumption. However, this was similar to the level of consumption recorded in the United Kingdom [10], the United States [11], Ireland [12] and Singapore [13], while other countries, such as France [14], Italy [15] and Malaysia [16] have far lower intakes. Scandinavian countries have recently increased intakes, in particular Denmark, where successful promotion of whole grains, via a public-private partnership, increased consumption from 33 g/day (from 2000 to 2004) to 55 g/day (in 2011–2014) [17].

Food Standards Australia New Zealand (FSANZ) define whole grain as the intact grain or the dehulled, ground, milled, cracked or flaked grain, where the constituents—endosperm, germ and bran—are present in such proportions that represent the typical ratio of those fractions occurring in the whole cereal, and includes wholemeal [18]. With the aim of proposing an international definition, the Whole Grain Initiative has suggested that whole grains shall consist of the intact, ground, cracked, flaked or otherwise processed kernel after the removal of inedible parts such as the hull and husk. All anatomical components, including the endosperm, germ, and bran must be present in the same relative proportions as in the intact kernel [19]. This definition closely aligns with the Australian definition. Defining whole grain foods is far more problematic and has been described as foods containing ≥51% whole grain by weight per reference amount customarily consumed by the United States Food and Drug Administration [20], and The Health Grain Forum has proposed that whole grain foods are ≥30% whole grain ingredients on a dry weight basis and contain more whole grain than refined grain ingredients [21]. Since FSANZ do not regulate claims describing the amount of whole grain in foods, a voluntary Code of Practice for Whole Grain Ingredient Content Claims designed to complement existing food standards and to assist consumers in meeting the 48 g Daily Target Intake (DTI) has been established by the Grains & Legumes Nutrition Council (GLNC). In order to gain an understanding of whole grain, GLNC had conducted attitude and behaviour surveys over a number of years and in 2017 results indicated that fifty percent of Australians were unaware of the definition of whole grain food [22]. Whole grain foods are commonly identified through the use of food labels, as well as by their appearance and colour. Research conducted outside of Australia has indicated that potential barriers to adequate whole grain consumption relate to taste, lack of understanding of health benefits, family influence, price and availability of whole grains [23,24]. Without sufficient knowledge of how to read a label and identify whole grains, consumers may become confused [25], or incorrectly mistake refined products for whole grain sources (e.g., multigrain bread), thereby unintentionally reducing whole grain intake. Therefore, understanding of consumers’ knowledge of whole grains may provide insight into strategies to increase whole grain consumption. The aim of this study was to conduct an online semi-structured questionnaire to investigate consumers’ knowledge, attitudes and identification of whole grains, incorporating an exploration of factors influencing consumption, promotion and provision.

## 2. Materials and Methods

A cross-sectional approach used an online survey (SurveyMonkey [26]) with both open and closed questions. Ethical approval for this study was provided by the University of Wollongong and Illawarra and Shoalhaven Local Health District Health and Medical HREC (Ethics number: 2019/049, Approval date: 26 February 2019). Consumer perspectives were sought from Australians over the age of 18 years, as adults are more likely to be responsible for their own food choices and parental consent was not possible for children. Individuals with formal nutrition qualifications or who were currently involved in nutrition education were excluded through the promotional materials for the survey due to the likelihood that this cohort would have greater knowledge associated with whole grains compared to the general population. Participants were alerted to this study through a combination of convenience, snowballing and purposive recruitment, via word of mouth, as well as advertisement on social media platforms such as Facebook, Twitter and Instagram via a nationwide promotion of the survey link. This study was also advertised in the GLNC newsletter to over 5000 subscribers. An incentive of a $50 grain food prize pack was offered to encourage participation.

Data were collected from March to May 2019. The survey (Appendix A) comprised 39 questions and was designed to gain information on general dietary information, consumption of grains, perceived whole grain definition, and, understanding and identification of ‘whole grains’. In addition to this, participants were asked whether they believed whole grains were important in their diet, if they were aware of the recommended daily intake of grains and whole grains, and any health benefits associated with their consumption. Questions associated with food labels and claims, including claims such as high in protein, contains whole grains, high in fibre and low glycaemic index (GI) were also included. The survey utilised multiple choice, check multiple choice box selection, rank and scale, a 3-point scale ranging from ‘never to frequently’, a 5-point scale ranging from ‘strongly agree to strongly disagree’, and free-text responses. Recorded demographic characteristics were age, gender, employment status, highest level of education, and residential area.

Questions in this study were modelled on a study conducted by McMackin et al. (2011) which utilised focus groups to gain in-depth information regarding consumer attitudes and awareness of whole grain as well as barriers and facilitators to their consumption [27]. Questions were refined in consultation with relevant stakeholders, and surveys piloted by five dietitians and three non-dietitians to assist with construct and content validity, as well as the general understanding of questions and flow of the survey tool.

Data were exported from SurveyMonkey to Microsoft Excel™ (Version 16.22, 2019, Washington, DC, USA), where data collation and cleaning occurred. Descriptive statistics were used to provide frequency counts and percentages for demographic information, multiple choice questions, check multiple choice box selection, rank and scale and Likert scale-related questions. Content analysis and themes were identified utilising data visualisation methods, including content or word clouds. These were used to identify, analyse and report patterns from the open-ended questions [28]. Content analysis of responses was used to explore topics and themes as they emerged in the data, representing a conventional approach to this analysis with no preconceived ideas as to the relevant themes derived from responses [29].

## 3. Results

### 3.1. Study Population

Sixty-one percent of surveys were completed in full, from 735 responses (*n* = 448). Partially completed questionnaires were included in the final analysis, as question responses were independent of one another. Most respondents were female (86.9%; *n* = 637), from two defined age groups 18–25 (23%; *n* = 168) or 36–45 years (23%; *n* = 168) and a larger proportion were employed full time compared with other employment categories (37.6%; *n* = 276). Most participants had achieved qualifications post high school of a certificate or diploma (37.4%; *n* = 274) or degree (26.6%; *n* = 195). Most commonly, participants stated that they lived in a regional area (42.4%; *n* = 311) and three-quarters (76.6%; *n* = 579) of participants were not following a specific diet (Table 1). Special diets were noted by 134 participants and included vegetarian/plant based (*n* = 37), gluten free (*n* = 32), low carbohydrate/ketogenic (*n* = 23), ‘for medical purposes’ (*n* = 22), and dairy free (*n* = 20).

Forty percent (*n* = 296) of respondents noted avoiding particular foods, including gluten/carbohydrates/bread/grains (29.4%; *n* = 87), dairy/lactose (26.7%; *n* = 79), sugar (14.5%; *n* = 43), meat (11.8%; *n* = 35) and processed foods (9.5%; *n* = 28).

### 3.2. Consumption of Grain Foods

Ninety-three percent of participants stated that they consume grain foods (*n* = 679/728). More than three-quarters of participants (*n* = 433/487) reported consuming less than the recommended serves of grains per day, with only 8% (*n* = 40) reporting quantities consistent with the recommended quantity for their age and gender. Over half (54%; *n* = 3) of males reported consuming 2–3 serves of grains per day (Table 2).

White pasta (43%; *n* = 283) was the most frequently purchased grain food followed by white rice (36%; *n* = 235) and whole grain bread (36%; *n* = 235). Participants chose to purchase whole grain varieties more frequently than refined/white varieties, with variability depending on the category of the food item. For breads, whole grain (wholemeal) was purchased more frequently than white varieties, at 36% compared to 26%, respectively. Within other grain varieties such as rice and pasta, refined varieties were purchased more frequently, with 36% and 43% of participants purchasing white rice and pasta, respectively, compared to 23% for brown rice and 12% for wholemeal pasta (Table 3).

Overall, 77.4% (*n* = 514/664) of participants reported consuming breakfast cereals, with a oat/porridge varieties most commonly consumed (*n* = 353/485), followed by muesli (*n* = 214/485), and granola (*n* = 153/485) (Table 4). Frequency of consumption of each cereal was not determined. Twenty-two percent of participants noted consumption of other breakfast cereals not listed, with the most commonly noted cereal being (whole grain) Weet-Bix™.

### 3.3. Grain and Whole Grain Knowledge and Awareness

Ninety-six percent of participants had previously heard of whole grain or whole grain foods (*n* = 489/507), although eighteen participants (3.5%) had never heard of them before. More than half of participants (62%; *n* = 393/635) were unable to comment on how many grain serves are recommended each day, although serve size information had been provided in an earlier question. When accounting for varying serve recommendations based on age and gender, only 7% (*n* = 44) of participants were able to correctly identify these recommendations. Similarly, more than half of participants (62%; *n* = 507) were unable to recall a recommended quantity of whole grain serves (from their grain serves), and only 9% (*n* = 46) correctly identified recommended serves based on their age and gender (Table 5).

Whole grains were commonly described by participants as a food made from a less processed grain (72%) or a food high in fibre (67%). Though these statements are true to an extent, less than half (49%) of participants correctly identified whole grains as specified by Food Standards Australia New Zealand, as a food that contains all components of the grain [18]. When open responses were utilised, almost half of participants (47%) stated that whole grains are less processed grains, with one-quarter (24%) of participants stating ‘whole grains are grains that used all its components’. Close to one-quarter (23%) of participants were unsure how a food might be classified as a whole grain.

Approximately half of the participants reported seeing whole grains mentioned on social media (*n* = 238/492) and television, respectively (*n* = 288/492). Others commented that they have also noticed mention of whole grains within cooking books, on blogs and menus. Yet, over half of the participants (59%; *n* = 298) have never had a health care professional mention whole grain to them. However, those that had most commonly note that they had heard the information from dietitians (25%; *n* = 126).

### 3.4. Participant Attitudes and Barriers

**Figure 1:**
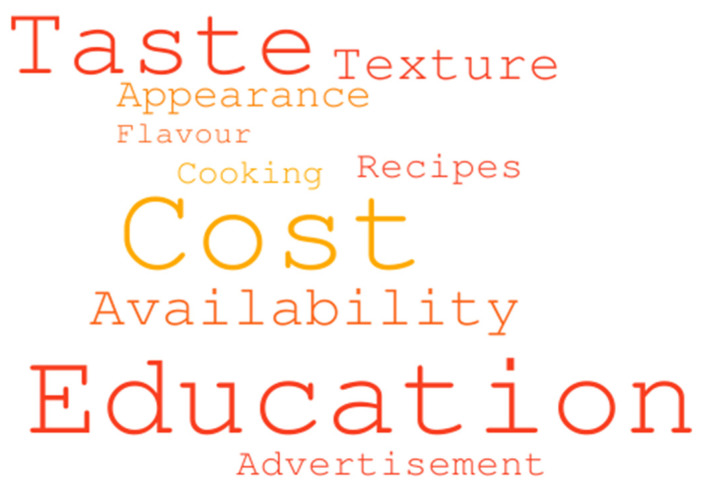
Word cloud based on responses to the question “What do you think could be done to help you choose whole grain foods more often?” Larger font indicates high frequency of mentions by participants.

Ninety-two percent (*n* = 468) of participants believed whole grains were important for their diet. Half of the very few participants (52%; *n* = 16) who did not believe that whole grains were important, stated that this belief was due to them being unaware of the importance of whole grains for health, and that no one had mentioned their significance. Common factors that participants reported help them choose whole grains foods more frequently included education, cost and taste (Figure 1).

### 3.5. Whole Grain Identification

**Figure 2:**
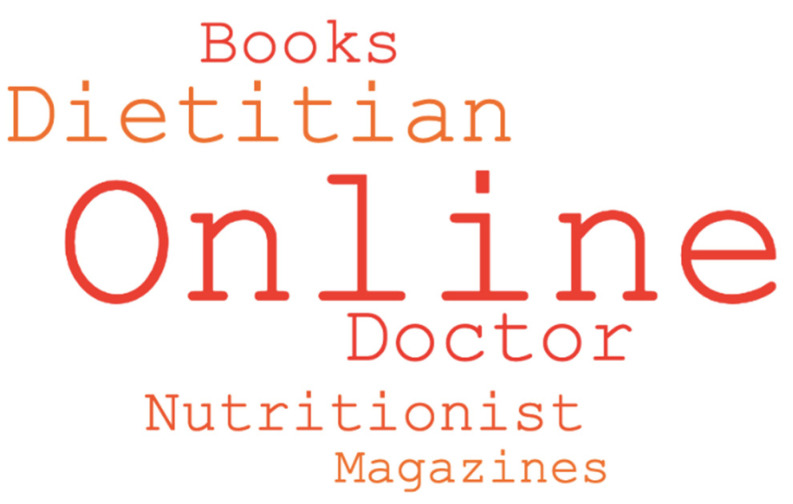
Word cloud based on responses to the question “Where would you go to seek nutritional information on whole grains?” Larger font indicates high frequency of mentions by participants.

Three times as many participants stated that they would seek information on whole grains via online sources (*n* = 320) compared to via qualified dietitians (*n* = 103) (Figure 2).

Almost all participants (93%; *n* = 418) had seen whole grains advertised on packaging in some form. However, consumers were less aware of the details or the amount of whole grain being claimed on packs such as “source or made of whole grains”. A whole grain food was most often identified via on-pack messaging (65%; *n* = 292) followed by the ingredients list (52%; *n* = 233). Fourteen percent of participants reported either not looking for whole grain items (14%; *n* = 62) or that they were unaware of what a whole grain food was (6%; *n* = 26) and were therefore unable to recall how they would identify whole grain foods.

When participants were asked to identify whole grain foods, they were most unsure about teff (65%; *n* = 343), spelt (48%; *n* = 243), black rice (38%; *n* = 191), red rice (52%; *n* = 260) and buckwheat (37%; *n* = 187) (Table 6).

### 3.6. Whole Grain Health Literacy

Seventy percent of participants reported that they were aware of positive health benefits associated with whole grains and the majority of participants were able to correctly answer true and false statements related to health benefits associated with whole grain consumption (Table 7). However, 43% of participants were most unsure about the ability of whole grains to decrease inflammation (43%; *n* = 218). Three-quarters of participants (75%; *n* = 381) stated that if they were further informed of the health benefits of whole grains, their opinion would change and they would be more willing to switch to whole grain options.

### 3.7. Whole Grain as an Ingredient

**Figure 3:**
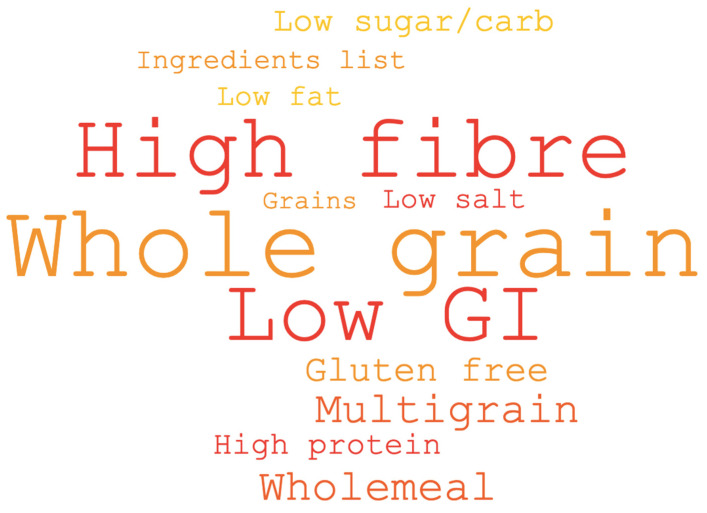
Word cloud based on open-ended responses to the question “Do you look for any specific words or phrases on packaging when purchasing the following items?” Items listed included bread, noodles, crackers, rice and pasta. Larger font indicates high frequency of mentions by participants.

When considering claims which may encourage purchasing decisions (Figure 3), ‘contains whole grains’ (18%; *n* = 83) was reported to be less important than high in fibre (34%; *n* = 153), high in protein (26%; *n* = 114) and low GI claims. (22%; *n* = 99). Despite this, when participants were asked to report the types of claims they commonly looked for on bread, noodles, crackers, rice and pasta, whole grain claims were most commonly referred to, followed by high fibre and low GI claims (Figure 3). The most common food item participants reported examining for specific phrases related to health was bread (56.5%; *n* = 253), with noodles being the least common (15.2%; *n* = 68).

Reasons whole grains were not considered to be important in the diet included “it was never mentioned to me”. The majority of participants stated that they were unsure why they were important, that nutrients found in whole grains could be found in other foods, and a number of participants reported following a ketogenic diet, which excludes all grain foods (Appendix B).

## 4. Discussion

This study provides some understanding of Australians’ views of whole grains, their attitudes, knowledge and health literacy and provides insights into factors that may affect choice of grain foods. Whole grain intake within Australia is below current recommendations [9] and one of the first steps in developing modes of action to increase intake is understanding Australians current knowledge related to whole grains, as this may impact food choices and the likelihood of making dietary changes.

While it must be noted that the sample was not representative of the Australian population, results from this study are consistent with other work suggesting that the participants consume less than the recommend amount of grain food serves per day for their age and gender. Data from the NNPAS 2011–2012 reported that 70% of Australians did not consume sufficient quantities of grains [8]. If individuals consume insufficient grains, then not only will they be likely to consume insufficient whole grains, but they may also be at risk of inadequate consumption of other key elements derived from grain foods such as dietary fibre, B group vitamins and iodine.

In order for individual adherence to dietary guidelines, an appropriate level of nutrition knowledge is required for success [30]. This study found that less than one in ten participants were able to correctly identify both the recommended number of grain serves per day and also recommended whole grain serves a day for their age and gender. Over half of all participants were unable to identify any recommended quantities of grain serves (62%) or whole grain serves (61%). This was an important finding, as without sufficient knowledge on whole grain recommendations, the success of positive health behaviours related to adequate intake is limited. In a study aimed at increasing fruit and vegetable intake, a nutrition intervention provided participants with information on nutrients in fruit and vegetables, the role these nutrients played in the body and current intake recommendations. At the end of this, 91% of participants within the intervention increased fruit and vegetable intake compared to only 22% in the control group [31]. This highlights the value in improved understanding of dietary guidelines and recommendations as a possible strategy to increasing knowledge, compliance and consumption.

Participants within this study primarily understood whole grains as a less processed grain, higher in dietary fibre. Only half of participants identified whole grains as a food containing all components of a grain when provided with multiple options, and even fewer participants noted this within open responses. Results were comparable to a previous attitude and behaviour survey, with 50% of participants not knowing precisely what constitutes a whole grain, but the majority acknowledging level of processing as a feature of these grain foods [22]. Findings from this study suggest that individuals may have difficulty in identifying whole grains and whole grain foods, particularly those described as wholemeal or those categorised as snacks or breakfast cereals, for example, wholemeal flour, popcorn and Weet-Bix™ or other cereal products. The possible basis for this misconception could be related to individuals believing whole grains are unprocessed or less processed grains [25]. Consequently, if they are transformed from their original “intact” form, they may no longer be perceived as whole grain by many consumers who assume a literal meaning for this food label. Correct identification of these manufactured whole grain foods could potentially encourage intake and therefore impact total daily whole grain consumption. The inability to differentiate whole grain and refined grain varieties is widely acknowledged as a barrier to consumption of grain foods [27,32]. Previous research on whole grains has identified barriers to whole grain consumption including texture and taste with a number of studies assessing the general acceptability of whole grain foods including muffins, bread, rolls, breakfast cereals, oats, muesli, pasta, brown rice, cookies, granola bars, cereal bars and other snacks [15,16,17,18]. Perhaps if individuals are informed that whole grain-containing foods come in a variety forms and they are able to identify these, preconceived taste and texture expectations may be overcome and consumption may increase.

The majority of participants (92%) believed that whole grains were important for their diet, as they provided dietary fibre and nutrients, they are less processed and more natural. However, almost all participants who stated the reverse, that whole grains were not important, explained that the reason for this belief was due to a lack of knowledge of the specific health benefits of whole grains. Overall, there were general perceptions of “healthfulness”, but thirty percent of participants were unaware of any specific health benefits associated with whole grains. The most frequent health benefits that participants associated with whole grains related to dietary fibre, digestion, bowel and gut health improvements and, that they are low GI. Digestive benefits are tangible for consumers. Recent work has noted that dietary fibre and whole grain content are better measures of carbohydrate quality than GI, so it is perhaps positive that fibre and broad health measures are most commonly recognised [33]. Few participants identified whole grains as important in reducing chronic illnesses such as cardiovascular disease, certain cancers and their potential risk factors—obesity, hypertension and elevated fasting glucose levels [2,3,4,5]. This indicates that individuals only have a basic understanding and awareness of whole grain health benefits, which is to be expected. It is unlikely that discussion of serious disease would take place in advertising or on social media, the key sources of information for participants in this study. Furthermore, making connections with serious disease via high-level health claims is not permitted on food product labelling.

Interestingly, three-quarters of participants stated that if they were informed of benefits associated with whole grain intake, it would encourage them to switch to whole grain options, which also corresponds with previous research [32]. Participants stated that being educated on identification of whole grains along with information on how to cook them would also act as facilitators to further consumption. A recently published paper increasing exposure to whole grain food choices among low whole grain consumers over six weeks was shown to improve ratings of liking, flavor, texture and willingness to include more whole grains in the diet [34]. This study emphasised that increasing exposure was key, with simple swaps rather than changes to the dietary pattern, leading to greater whole grain intake. A previous study utilised nutritional lectures to educate college students on physiological benefits of whole grains in relation to disease prevention, along with enabling them to identify whole grain foods on food labels and become involved with tasting activities. The study aimed to reduce taste and texture biases, and resulted in an increase in whole grain intake from 10% to 38% of total grain intake [35]. Generally, multi-pronged approaches, with education and tasting, produce the outcomes required to impact dietary change.

Individuals in this study reported using on-pack messages to identify whole grain foods. While FSANZ do not regulate the use of on-pack whole grain claims, as they do with other nutrients, such as dietary fibre, protein, carbohydrate, and fat, the voluntary Code of Practice for Whole Grain Ingredient Content Claims in Australia, or overseas, labelling using whole grain “stamps” is used to guide consumers [36,37]. According to research that aimed to assess the impact of the GLNC Code, the use of the on-pack claims was more prevalent in certain food product categories, with 75% of breakfast cereals and breads using the specified claims compared to just 40% of crispbreads/crackers/rice and corn cakes and rice, pasta, noodles or other grains [36]. Although there was a high prevalence of whole grain claims on pack labels, it was estimated that only 43% of all eligible Australian grain food manufacturers and 65% of eligible grain food products in Australia are registered with the Code [36]. Here, less than one-quarter of participants reported looking for whole grain foods specifically and almost half never looked for the term ‘whole grains’ on packaging. In research focused on multi-attribute choice modelling using bread as the example, Burke et al. [38] found that whole grain was rated highly for both healthful and value for money by consumers. If more manufacturers routinely declared the percentage of whole grain content on packs, there would be a wider range of whole grain products for consumers to easily identify, providing the greatest opportunity to improve consumption patterns of whole grain foods across the population.

Aligned with this, strengthening public policy and regulation through the government, health professionals, health organisations and the food industry, perhaps via successful public–private partnership models utilised elsewhere, will become increasingly important to encourage whole grain and whole grain food consumption [39]. Key to this research and the promotion of whole grains is the concept of health, nutrition and food literacy. Health literacy, as defined by the World Health Organisation, is “the cognitive and social skills which determine motivation and ability of individuals to gain access to, understand and use information in ways which promote and maintain good health” [40]. Food literacy can be defined as the everyday practicalities associated with navigating the food system and being able to use it to ensure a food intake that aligns with nutrition recommendations [41]. While there may be some ambiguity in these definitions, they are important in nutrition research because they can assist in determining how a consumer’s knowledge may affect their health [42,43]. This is relevant when choosing between refined and whole grain foods. In order to assist Australians in making healthier choices, the Health Star Rating (HSR), a voluntary front-of-pack (FOP) labelling system, was adopted in Australia and New Zealand in 2014. Like other systems used internationally, the HSR was introduced as a strategy to guide selection of healthier foods in an easily understood manner, ranking foods on a scale between 0.5 (1/2) and 5 stars, based on the premise that more stars equate to a healthier product [44]. The HSR system was designed to reflect principles outlined in the Australian Dietary Guidelines, and provide consumers with a simple measure to compare foods within the same category [45]. The current HSR does not include whole grain within the algorithm. Some whole grain foods achieve a higher HSR due to the positive points gained from dietary fibre. However, research demonstrates that there is less than one star difference across multiple grain food categories [46]. Addition of whole grains to the algorithm may assist in promotion of their intake.

Diets with varying carbohydrate content and aversions to carbohydrate foods and grains have gained substantial popularity in recent years [47,48]. This was evident within this study, with almost all reasons individuals provided for not consuming whole grains related to participants’ dietary beliefs and regimens, with gluten-free (not for medical purposes), ketogenic, paleo and low-carbohydrate diets mentioned. Though diets lower in carbohydrate have been shown to have short-term weight-loss benefits, the long-term effects and safety of these diets are not thoroughly studied or understood [49,50]. These diets exclude major carbohydrate food sources such as whole grain breads, pasta, and starchy vegetables including potatoes and corn, resulting in a significant decrease in carbohydrate, dietary fibre and micronutrient intakes of non-haem iron, magnesium, folate, thiamin, and iodine, which are the key nutrients supplied predominantly by grain foods in the Australian diet [51]. With the decreased intake of these nutrients due to elimination of their primary sources, potential gastrointestinal consequences such as constipation, increased risk of bowel cancer [52,53] and altered gut microbiota composition [54] may impact overall health. This provides further evidence of the importance of education on the benefits of whole grain foods to assist in removing the negative stigma that implies that all carbohydrate sources are ‘bad’.

Online surveys are beneficial to reach a large array of individuals in a relatively short period [55,56]. However, there may be unintentional exclusion of individuals, particularly those in remote areas, or of lower socioeconomic status or those of an older age. Although an active attempt was undertaken to ensure that a representative population was achieved for this study through active promotion via social media, the use of convenience sampling resulted in a less generalisable population, and an especially high proportion of female participants (87%), who tend to have higher food literacy. Importantly, the survey targeted the age groups making up ~65% of the Australian population [57]. Although there were a limited number of participants ≥65 years of age, NNPAS data suggest that those ≥71 years of age already consume more whole grain than any other age group within the population (33.7 g/d) [9]. Furthermore, of the 735 participants who commenced the survey, only 448 completed it, potentially due to the length of time required for completion. It was also possible that participants completing the survey may have had a greater general interest in nutrition compared to the general population. It is important to note that there was some unintentional ambiguity in the question asking consumers to classify multigrain and grainy bread with respect to whole grain content. Multigrain breads tend to be made from refined flour with the addition of seeds and grains, and therefore not classified as a whole grain food. However there are exceptions and some multigrain breads may be made from wholemeal (whole grain) flours and as a result participants’ responses may have varied depending on the type of multigrain and grainy breads encountered. Furthermore, consumption data received from this study were self-reported and based on estimates by participants and the precise amounts of grain food intake were not quantified. Therefore, consumption estimates can only be use as an indication of grain intake and not as an exact measure.

## 5. Conclusions

This study aimed to investigate consumers’ knowledge, attitudes and identification of whole grains, with an exploration of factors influencing consumption, accessibility, promotion and provision. Findings highlighted the necessity of education on whole grain health benefits. For consumers, improving health literacy, by providing ideas on how to identify whole grains and whole grain foods, and practical ways for individuals to incorporate these foods into their current lifestyle are needed. Furthermore, promotion of whole grains and the increased use of prominent and clear labelling of whole grain in foods may be beneficial to clearly identify all possible categories of products. Finally, removing the negative stigma associated with carbohydrate foods, including grains, will be necessary to improve consumption and facilitate active choices to incorporate the best food options. While health professionals like dietitians may be best placed to provide information to consumers, proportionately, very few within the population access their services. More broad-reaching campaigns may be required to make more sustainable changes in consumption, leveraging the support of the government, regulators, the food industry and non-government organisations within a public–private partnership model.

## Figures and Tables

**Table 1:** Demographic characteristics of participants.

Demographic Variable	Frequency (%)
*Gender (n* = *733)*	
Male	93 (12.7)
Female	637 (86.9)
Prefer not to answer	3 (<1%)
*Age in years (n* = *733)*	
18–25	168 (22.9)
26–35	137 (18.7)
36–45	168 (22.9)
46–55	142 (19.4)
56–65	82 (11.2)
65+	36 (4.90)
*Employment status (n* = *734)*	
Employed, working full time	276 (37.6)
Employed, working part time	140 (19)
Employed, casual	78 (10.6)
Not employed	52 (7.1)
Retired	54 (7.4)
Student	62 (8.5)
Unable to work	25 (3.4)
Prefer not to answer	11 (1.5)
*Education status (n* = *733)*	
Some high school	81 (11)
Completed high school	125 (17)
Certificate/diploma	274 (37.4)
Bachelor’s degree	195 (26.6)
Master’s degree	52 (7.1)
PhD/doctorate	6 (<1%)
*Residential area (n* = *734)*	
Rural	149 (20.3)
Remote	9 (1.2)
Regional	311 (42.4)
Inner city	36 (4.9)
Urban area	156 (21.3)
Metropolitan area	73 (10)
*Diet (n* = *727)*	
No specific diet	579 (76.6)

**Table 2:** Reported daily grain serve consumption of participants.

Serves	Frequency (%)	
*Serve of Grains Consumed Each Day (n* = *487)*	Female *(n* = *421)*	Male *(n* = *63)*
**1**	56 (13.3)	9 (14.3)
**2**	124 (29.5)	16 (25.4)
**3**	104 (24.7)	18 (28.6)
**4**	86 (20.4)	10 (16)
**5**	34 (8)	6 (9.5)
**6+**	17 (4)	4 (6.4)

**Table 3:** Consumers frequency of grain purchases ^1,2^.

Grain Foods	Frequency (%)			
	Never	Sometimes	Frequently	*n* Total
Oats	30 (13.7)	**365 (55.7)**	200 (30.5)	655
Brown rice	146 (22.4)	**355 (54.5)**	150 (23)	651
White rice	63 (9.6)	**359 (54.6)**	235 (35.8)	657
Wild rice	**459 (71.7)**	168 (26.3)	13 (2)	640
Black rice	**492 (77)**	135 (21)	12 (2)	639
Red rice	**575 (90)**	58 (9)	5 (0.8)	638
White pasta	67 (10)	**307 (46.7)**	283 (43)	657
Wholemeal pasta	244 (37.7)	**325 (50)**	78 (12)	647
White wraps	196 (30.5)	**359 (55.8)**	88 (13.7)	643
Wholemeal wraps	186 (28.8)	**366 (56.6)**	95 (14.7)	647
Whole grain wraps	223 (34.7)	**309 (48)**	111 (17.3)	643
Whole grain bread	142 (21.8)	**274 (42)**	235 (36)	651
Wholemeal bread	202 (31.3)	**289 (44.8)**	154 (24)	645
White bread	210 (32.7)	**269 (41.8)**	164 (25.5)	643
Multigrain bread	125 (19.3)	**307 (47.3)**	217 (33.4)	649
Wholemeal flour	**319 (49.8)**	268 (42)	53 (8.3)	640
White flour	106 (16.3)	**362 (55)**	183 (28)	651
Enriched flour	**566 (89)**	61 (9.6)	9 (1.4)	636
Popcorn	161 (25)	**357 (55)**	130 (20)	648
Quinoa	**291 (45)**	290 (45)	64 (10)	645
Buckwheat	**515 (80)**	110 (17)	18 (2.8)	643
Rye	**450 (70)**	167 (26)	26 (4)	643
Spelt	**550 (85.7)**	81 (12.6)	11 (1.7)	642
Teff	**611 (91.7)**	29 (4.5)	0	640
Freekeh	**587 (91.7)**	45 (7)	8 (1.3)	640
Bulgur	**562 (87.7)**	74 (11.5)	5 (0.8)	641
Barley (pearled)	**405 (63)**	226 (35)	12 (1.9)	643
Triticale	**630 (97)**	16 (2.5)	2 (0.3)	638
Sorghum	**589 (92.3)**	44 (6.9)	5 (0.8)	638
Millet	**584 (91.7)**	48 (7.5)	5 (0.8)	637
Rice noodles	178 (27)	**400 (61)**	76 (11.6)	654
Wheat noodles	**367 (57.5)**	240 (37.6)	31 (4.9)	638
Whole grain noodles	**480 (75)**	145 (22.7)	15 (2.3)	640
Muesli or oat bars	174 (26.8)	**352 (54)**	124 (19)	650
Plain crackers	83 (12.8)	**406 (62.4)**	162 (24.9)	651
Whole grain crackers	169 (26)	**355 (55)**	120 (18.6)	644

^1^ Whole grains and whole grain-containing foods are underlined. ^2^ Highest frequencies are bolded.

**Table 4:** Frequency of breakfast cereals selected while shopping.

Breakfast Cereals *(n* = *485)*	Frequency (%)
Kids cereals	128 (26.4)
Muesli	214 (44)
Granola/clusters	153 (31.6)
Oats/porridge	353 (72.8)
Other	107 (22)

**Table 5:** Recommended number of grain and whole grain serve per day as identified by participants.

Serves	Frequency (%)	
	*Grain Serves (n* = *635)*	*Whole Grain Serves (n* = *507)*
1	14 (2.2)	14 (2.8)
2	38 (6)	41 (8)
3	65 (10.2)	44 (8.7)
4	48 (7.6)	48 (9.5)
5	51 (8)	30 (5.9)
6+	26 (4)	19 (3.8)
unknown	393 (62)	311 (61.3)

**Table 6:** Participants’ classification of foods as whole grain or whole grain containing ^1,2^.

Grain Foods	Frequency (%)			
	Not a Whole Grain	Whole Grain Containing	A Whole Grain	Unsure
Rye	26 (5)	75 (15)	**276 (55)**	123 (25)
Teff	9 (2)	23 (4.5)	**128 (25.5)**	343 (68)
Oats	43 (8.5)	119 (24)	**283 (56)**	59 (12)
Spelt	15 (3)	49 (10)	**195 (39)**	243 (48.4)
Quinoa	16 (3)	65 (13)	**292 (58)**	130 (26)
Popcorn	112(22)	114 (23)	**155 (31)**	123 (24.4)
Weet-Bix™	82 (16)	**302 (60)**	54 (9)	76 (15)
Wild rice	12 (2)	59 (12)	**295 (58)**	140 (28)
White rice	**238 (47.5)**	76 (15)	87 (17.4)	100 (20)
Brown rice	22 (4.4)	113 (22)	**281 (56)**	88 (17.5)
Black rice	15 (3)	64 (13)	**235 (47)**	191 (38)
Red rice	17 (3.4)	50 (10)	**174 (35)**	260 (52)
Vita-Weats™	65 (13)	**325 (65)**	28 (5.5)	88 (17.4)
Ryvita’s™	59 (12)	**278 (55.4)**	30 (6)	135 (27)
Buckwheat	10 (2)	47 (9.4)	**257 (51)**	187 (37)
Rice cakes	**186 (35)**	173 (34.4)	16 (3)	128 (26)
Brown rice cakes	59 (12)	296 (59)	**26 (5)**	119 (24)
Wheat pasta	**176 (35)**	174 (35)	23 (4.6)	131 (26)
Wholemeal pasta	76 (15)	289 (58)	**32 (6.4)**	104 (21)
White wrap	**356 (71)**	46 (9)	9 (1.8)	91 (18)
Wholemeal wraps	104 (21)	**284 (57)**	25 (5)	90 (18)
White bread	**377 (75)**	41 (8)	3 (0.6)	80 (16.3)
Grainy bread	**37 (7.3)**	358 (71)	34 (6.75)	75 (15)
Wholemeal bread	99 (20)	**296 (59)**	25 (5)	79(15.8)
Multigrain bread	**27 (5)**	376 (74)	49 (9.7)	54 (11)
Rye bread	67 (13)	**280 (56)**	32 (6.4)	124 (25)
Sourdough	**261 (52)**	82 (16.4)	12 (2.4)	146 (29)
White flour	**366 (73)**	32 (6.4)	7 (1.4)	96 (19)
Enriched flour	**221 (44)**	73 (15)	7 (1.4)	199 (40)
Wholemeal flour	106 (21.2)	259 (52)	**42 (8.4)**	94 (18.8)
Rice noodles	**245 (49)**	91 (18)	12 (2.4)	151 (30.3)
Wheat noodles	**189 (38)**	152 (30.5)	13 (2.6)	145 (29)
Whole grain noodles	49 (10)	**319 (64)**	36 (7)	97 (19.4)
Plain crackers	**323 (65)**	47 (9.4)	5 (1)	125 (25)
Whole grain crackers	47 (9)	**345 (69)**	38 (7.6)	72 (14.3)

^1^ Whole grain foods are underlined; ^2^ correct answers are bolded.

**Table 7:** Participant perceptions of whole grain associated health benefits ^1^.

Survey Question	Frequency (%)		
*Health-Associated Whole Grain Statements (n* = *507)*	True	False	Unsure
Causes weight gain	32 (6.3)	**405 (80)**	70 (14)
Causes inflammation	37 (7.3)	**366 (72)**	104 (21)
Decreases inflammation	**222 (43.8)**	67 (13)	218 (43)
Lower risk of bowel cancer	**396 (78)**	19 (4)	92 (18)
Better weight maintenance	**388 (76.5)**	29 (6)	90 (17.5)
Improves cholesterol	**359 (70.8)**	24 (5)	124 (25)
Reduces risk of heart disease	**351 (69)**	19 (4)	137 (27)
Reduces risk of type 2 diabetes	**340 (67)**	26 (5)	141 (27.8)
Healthier waist measurements	**280 (55)**	42 (8.3)	185 (36.5)
Causes spike in blood sugar levels	34 (6.7)	**344 (68)**	129 (25.4)
Helps keep you feeling fuller for longer	**453 (89.4)**	11 (2)	43 (8.5)

^1^ Correct answers are bolded.

## Appendix A

Survey Questions

1. What is your age group?
2. What is your gender?
3. Which of the following categories best describes your employment status?
4. What is the highest level of education you have completed?
5. How would you describe the area you live?
6. Do you follow a specific diet? (For example, vegetarian, gluten free, medical condition)
7. Do you avoid any particular foods?
8. Do you consume grain foods?
9. Please tick what items you purchase from the following. (Never, Sometimes, Frequently)
10. Do you consume breakfast cereals? (including muesli/granola/oats/porridge/flakes)
11. In the breakfast cereal aisle what foods do you look for? (tick any that apply)
12. Please specify what breakfast cereal types/brands you consume. (open box)
13. Based on the above information on average how many **grain food** serves do you consume per day? (0–6+)
14. The Australia Dietary Guidelines provides recommendations for the number of serves individuals are supposed to consume. Are you aware of how many **grain food** serves are recommended for you (for your age and gender) per day? (1–6 and no)
15. Have you heard of whole grains or whole grain foods?
16. What do you think whole grains are? (Open question—“unsure” is an acceptable answer)
17. What is your understanding of the term “whole grain”? (tick as many as you want)
18. Have you seen whole grains mentioned on any of the following? (tick all that apply)
19. Have whole grains been mentioned to you by any of the following health care professionals? (tick all that apply)
20. Do you believe wholegrains are important to your diet? (yes/no)Please specify. (open box)
21. If a product says it is made of whole grains, how processed do you believe it is? (Likert scale with natural to processed—drag the dot)
22. Are you aware of how many serves of **whole grain** foods are recommended for you per day? (options 1–6 and no)
23. Are you aware of how many serves of **whole grains** are recommended for a child per day?
24. From the following foods, which foods do you believe are whole grains or whole grain containing foods? (Three columns: not a whole grain, whole grain, whole grain containing)
25. Are there any reasons why you would not choose whole grain foods when shopping? (open box)
26. Are you aware of any positive health benefits associated with consuming whole grains? (yes/no)Please specify. (open box)
27. From the following please specify if you believe the statements to be true or false about whole grains. (true, false, unsure)
28. Would being informed that whole grains are good for you change your opinion on them?
29. Have you heard of grains being discussed in a negative way? For example, friends choosing grain free food options.
30. Where would you go to seek nutritional information about whole grains? (For example: the internet, books, magazines, doctors, nutritionists, dietitians)
31. What do you think could be done to help you choose whole grain foods more often? (For example: taste, cost, texture, availability, education on whole grain foods, appearance)
32. When shopping you may see the following claims on food packaging. Rank what is the most important to you when purchasing food items. (1 being the most important and 4 being the least)
33. Have you seen any of these statements on food items when shopping? (Tick all that apply)
34. Do you regularly look for whole grain claims on packaging when you are shopping? (tick)
35. Do you actively choose foods that say they contain whole grains over foods that do not? (Likert scale, 0 to 100%)
36. How do you identify if a food is a whole grain food or not? (Tick all that apply)
37. Do you look for any specific words or phrases on packaging when purchasing the following items? For example: low GI, high fibre, contains whole grains. (Tick, if ticked asked to specify in open box below)
38. How much do you agree with the following statements about why you eat whole grains/whole grain foods? (Strongly agree, agree, neutral, disagree, strongly disagree)
39. Are you aware of the Grains and Legumes Nutrition Council?

## Appendix B

**Table A1:** Consistent themes emerging from the data and keywords that summarise participant responses related to whole grain knowledge, attitudes, consumption and health literacy, in descending frequency.

Theme	Subthemes/Keywords	Exemplar Quotes
Whole grains	Unprocessed	“Unprocessed grains”
	Natural	“Natural”
	Whole grain	“Includes the whole grain”
	Unsure	“I don’t know”
	Containing husk	“Grain that contains all its components”
	Not crushed/ground	“Grains that are still whole, not crushed up”
	Seeds	“Grain food with seeds”
Diet importance	Fibre	“Good source of dietary fibre”
	Nutrients	“Provide nutrients”
	Less processed	“The food that have the least processing are better for you”
	Natural	“They’re the most natural, best for you”
	Unsure	“I heard they are good for you but unsure why”
	Healthier choice	“Seems like a healthy choice”
	Fuller for longer	“Appetite control”
	Digestion	“Good for the digestive system”
	Bowel health	“Keep you regular”
	Gut health	“It is important for gut health”
	Low GI	“Some have a low glycaemic index”
	Healthy diet	“Important part of a balanced diet”
Health benefits	Fibre	“Higher fibre”
	Gut health	“Improves gut health”
	Digestive health	“Good for digestion”
	Low GI	“Lower GI so good for combating diabetes”
	Regularity	“To assist in regular bowel movements”
	Nutrients	“More nutrition content than processed grains”
	Lowers cholesterol	“Lower cholesterol, better for heart”
	Fuller for longer	“Keep fuller longer and better for you”
	Heart health	“Reduce risk of heart disease”
	Blood sugar control	“Helps control blood sugar levels”
	Reduced diabetes risk	“Reduce risk for diabetes”
	Lower cancer risk	“Lower bowel cancer risk”
Barriers to purchasing	Taste	“Worried about lack of taste”
	Price	“If they are priced considerably more than traditionally processed foods”
	Family influences	“My family not liking the taste. If it wasn’t for them I’d buy them”
	Texture	“Texture in things like bread and wraps”
	Dietary requirements	“We are coeliac and eat gluten free”
	Culinary use	“Compatibility with food dishes that I am cooking”
	Diet choice	“Rarely eat foods containing gluten”
	Cooking	“Unsure how to cook some of the rarer ones”
Negative grain association	Gluten-free diet	“Many people seem to think a gluten free diet is healthier”
	Weight gain	“Weight issues and paranoia about gluten fattening, I’ve cut out carbs at dinner”
	Taste	“Husband says grains taste like bird seed”
	Carbohydrates are bad	“Too many carbs are meant to be bad for you”
	Paleo diet	“Friends following paleo diet”
	Keto diet	“Carb free diets and keto”
	Fad diets	“People who are dieting avoid them”
